# Development of a Low Cost Assistive Listening System for Hearing-Impaired Student Classroom

**DOI:** 10.1155/2013/787656

**Published:** 2013-05-23

**Authors:** Setha Pan-ngum, Tharapong Soonrach, Sangvorn Seesutas, Anukool Noymai, Pasin Israsena

**Affiliations:** ^1^Department of Computer Engineering, Faculty of Engineering, Chulalongkorn University, Phyathai Road, Patumwan, Bangkok 10330, Thailand; ^2^National Electronics and Computer Technology Center, Rehabilitation Engineering and Assistive Technology Institute, 112 Thailand Science Park, Khlong Luang, Pathumthani 12120, Thailand

## Abstract

This paper describes the design, development, and tests of a low cost ALS. It was designed for hearing-impaired student classrooms. It utilised digital wireless technology and was aimed to be an alternative to a popular FM ALS. Key specifications include transmitting in 2.4 GHz ISM band with eight selectable transmission channels, battery operated and chargeable, pocket size, and ranged up to thirty metres. Audio characteristics and user tests show that it is comparable to a commercial system, currently employed in our partner school. The results also show that wearing an ALS clearly improves hearing of hearing-impaired students. Long-term usage by school children will be monitored to evaluate the system robustness and durability.

## 1. Introduction 

Assistive listening systems (ALSs) are devices that help hearing-impaired people, by reducing the effects of ambient noise and distance on sound travel. Sound progresses from a source, such as a speaker, through air to listener's ears. During travel, sound quality deteriorates. Longer distance reduces amplitude, and surrounding noise interferes with the original sound. This combination causes hearing difficulty to normal listeners, and the effects are more significant to hearing-impaired people.

An ALS generally consists of a transmitter and a receiver. The transmitter picks up sound near the source, usually by a microphone. It then converts it into electronic signal. The signal is transmitted to the receiver through wires, or by wireless transmission protocols. The receiver sends the signal directly to a hearing aid or converts it back to sound for the user. Since the signal is transmitted electronically, the adverse effects of distance and background noise are eliminated. The sound quality that a listener hears is close to the sound quality from the sound source. Hence, an ALS essentially maintains a signal-to-noise ratio (SNR) of the sound source. ALS could be used together with a hearing aid. The hearing aid function is to amplify the sound to compensate for a user's hearing impairment.

A hearing-impaired person has difficulty differentiating the sound source from surrounding noises [1]. ALS helps hearing-impaired people to participate in social activities with normal-hearing people, such as in classrooms or living rooms. For example, by placing a transmitter near to a television, a hearing-impaired person can watch TV programs with normal-hearing family members. Without ALS, the hearing-impaired person would have to turn up TV volume so much that other family members might find it disturbing. 

Signal transmission between transmitter and receiver through wire obviously limits ALS practical use and portability. Personal ALSs, therefore, rely on wireless transmission. Current ALSs employ one of these three technologies: induction loop (IL) [2], infrared (IR) [3], and frequency modulated (FM) radio systems.

Of the three systems, FM is the most popular. The sound signal is frequency modulated onto a radio-frequency carrier wave. The wave is sent from a transmitter to a receiver, which is set to the same frequency. The receiver then demodulated the carrier wave to retrieve the original sound signal. The technology is the same as radios, where radio stations are transmitters and household radios are receivers. 

**Figure 1:**
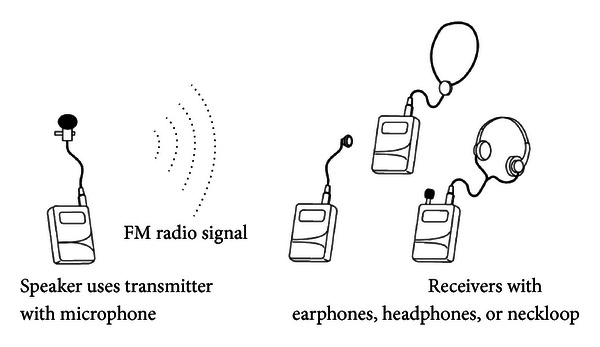
An example of an FM system [4].

The original sound signal can reach the user by earphone, headphone, or through a hearing aid via a neckloop or digital audio input (DAI) connector. An example FM system from Williams Sound Corporation is shown in Figure 1 [4].

FM systems were initially employed in broadcasting applications, such as in classrooms for hearing-impaired students [5]. Current advance in microelectronics enables robust and portable FM systems to be used as a standalone, point-to-point personal device. For instance, a user can place a portable transmitter near a television set. With the receiver connected to a hearing aid, the user can enjoy watching TV programs with the best SNR for sound.

As with FM radios, FM ALSs have to operate within approved frequency bands. The United States Federal Communications Commission (FCC) allocated 72–76 MHz frequency band for ALS usage. Subsequently, 216-217 MHz was also allowed for ALS. However, there is no specific frequency band for ALS globally. 

FM ALS has advantages over the IL system in terms of lower transmitting power and longer range. It also requires no line of sight between transmitter and receiver, as compared to IR. However, FM ALSs can be subject to signal interference and bad reception. FM radio users probably have experienced similar problems, which adversely affect sound quality. 

For the above reasons, this paper describes our development of a low-cost wireless ALS broadcasting system. The system utilises a wireless digital technology communication. It operates in a 2.4 GHz unlicensed industrial, scientific, and medical (ISM) radio band. The internationally unrestricted ISM band allows the system to operate in most countries. Due to the band-wide ranging applications, ISM-based components are readily available and at low costs. Furthermore analogue FM signal could be digitally processed to reduce noise and improve signal quality. The system is designed to be short ranged and in one-to-many broadcasting mode. It is intended to be used in a classroom for the hearing-impaired students. 

This paper is organised into four sections.  Section 1 contains the introduction.  Section 2 describes system specifications and architecture.  Section 3 reports system implementation. Acoustic performance and field tests, results, and analysis are also described in Section 3. The last section comprises conclusions.

## 2. System Specifications and Architecture

### 2.1. System Specifications

This project has produced three previous versions of the system, before achieving the final set. The earlier versions were assembled on development boards, temporary boxes, and field cases. The assembly and test results were reported in [6]. The analysis and user feedbacks from those results were used to design and assemble the final system. 

For practical classroom of the hearing-impaired usage, the system was designed to meet the following specifications:

- transmits in broadcast mode at 2.4 GHz,
- uses chargeable battery that lasts at least 4 hours (half-day usage),
- is chargeable via portable charge switching (same as mobile phone charging),
- can work with both internal and external microphones,
- has eight selectable frequency channels,
- has working range of up to 30 metres,
- has to fit into a shirt pocket and could be tucked onto a belt.

As mentioned earlier, the 2.4 GHz is the international frequency band, allocated for scientific and medical usage. A device can transmit in this band without having to seek government permission. 

The partner school currently uses commercial FM systems. Teachers carry transmitters and students carry receivers with them during class. Students return their devices to teachers during lunch and at the end of school day for charging. Hence, the specifications are designed to meet their usage, in terms of ease of charging and battery life.

In class, teachers speak into an external microphone which is connected to the transmitters. However, internal microphone was added to the specification for flexibility of usage. For example, a transmitter can be placed in front of a television or radio. In this case, an external microphone would not be needed.

Selectable frequency channels were included to avoid interference from nearby classrooms. When interference occurs, a teacher can tell all students to switch to different channels. 

Normal classroom length is six to eight metres. The 30-metre operating range is placed in the specifications to meet a teacher's suggestion that it should be long enough to cover outdoor class.

For practical purpose, the system has to be portable. Students put them in their shirt pockets, and teachers tuck them on their belts; so our system size was designed to meet this requirement.

### 2.2. System Architecture

**Figure 2:**
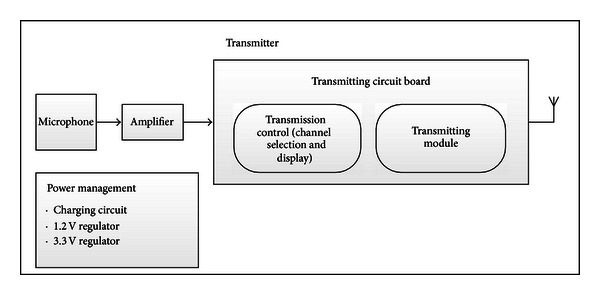
Transmitter module.

From the above specifications, the system architecture was designed as shown in Figure 2.

The signal from a condenser microphone was modified with an analogue amplifier circuit. The amplified signal was sent to a dsPIC33FJ128GP708A 16-bit low power microcontroller [7]. The microcontroller was connected to a user channel selector switch. It then sent control signal to the transmitting module accordingly. All user switches and displays such as status LEDs and seven-segment LED for channel display were controlled by this microcontroller. Transmission was done by U3 TX-Audio-2.4/AE processor, which applied frequency shift keying (FSK) modulation [8]. The processor could transmit at eight different frequencies between 2400 and 2483.5 MHz.

The power management circuit consisted of analog charging circuit and the 1.2 V and 3.3 V regulator circuits.

**Figure 3:**
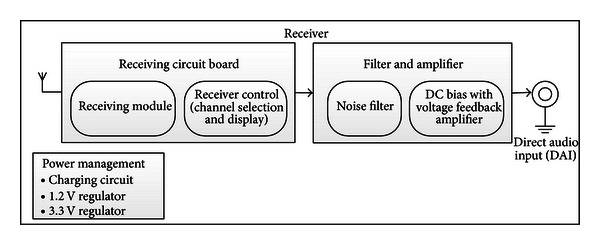
Receiver module.

The receiver module is shown in Figure 3.

The receiving circuit board contained the same microprocessor and microcontroller as the transmitting circuit board. They performed the reverse tasks, by basically selecting the channels and demodulating signal. Before reaching a listener, the signal was improved by filtering noise and amplified. Filtering was done by a simple low pass filter. The amplifier circuit output was adjusted by user rolling switch. The user could then select desirable output volume. The power management circuit was the same as in the transmitter.

## 3. System Implementation and Testing

### 3.1. System Implementation

**Figure 4:**
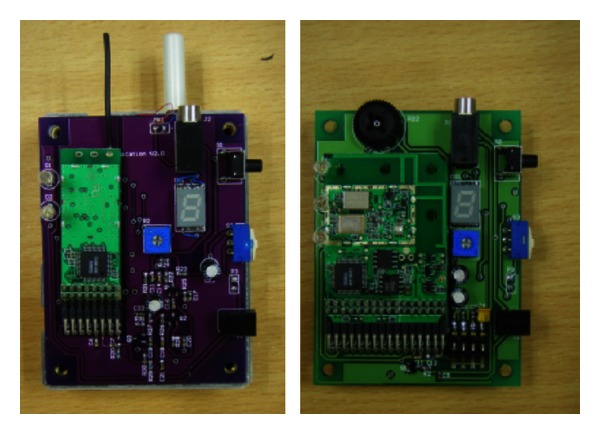
Transmitter and receiver PCBs.

**Figure 5:**
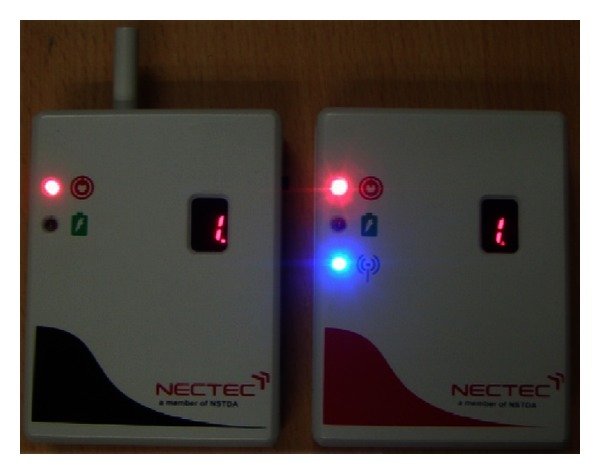
Transmitter and receiver casing.

Since the system was designed for actual usage by primary school children, robustness and durability were the main concerns. All the circuits were integrated into a single two-sided printed circuit board (PCB). Moderately thick PVC casing was used to house the assembly. The case had width, length, and thickness 2.7 centimetres, 7.8 centimetres, and 2.7 centimetres, respectively. The transmitter and receiver PCBs are shown in Figure 4. The finished transmitter and receiver in casing are displayed in Figure 5.

### 3.2. System Testing

Performance characteristics and user tests were done on the system to evaluate its performance.

#### 3.2.1. Performance Characteristics Test

 The system was tested for three key performance characteristics prior to the field test on users. They were the full-on gain, measured via HFA FOG 50 dB setup following IEC60118-7 standard, total harmonic distortion (THD), and its audio bandwidth. These parameters would help us determine the quality of audio signal produced, as the ALS was designed with a built-in amplifier. Full-on gain will tell us the amplification gain, bandwidth the range, and THD the quality of the signal. 

**Figure 6:**
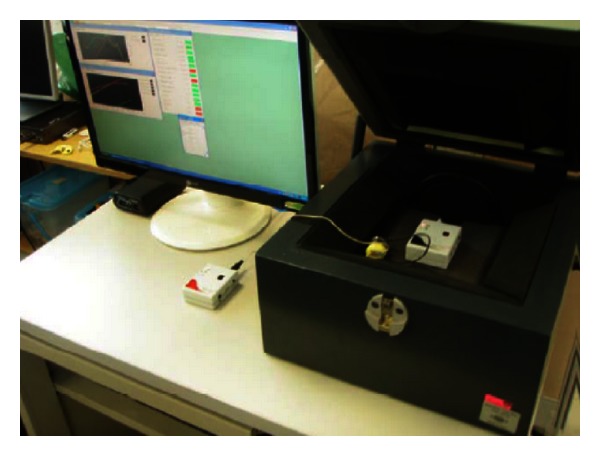
Performance characteristic test setup.

 The test was done using the software “Sound Check 8.1” and Anechoic Test Box Type 4232. The transmitter was placed in the box next to a speaker, which played a sound at different test frequencies. The receiver was placed next to the box. The received signal from the receiver was passed on to the box microphone. The signal picked up from the microphone was sent to the software for evaluation. The experiment setup is shown in Figure 6.

The software calculated HFA FOG 50 dB, which is the high frequency average gain given input at 50 dB, audio bandwidth, and total harmonic distortion (THD). 

The HFA FOG 50 dB was 67.3 dB. The audio bandwidth was the range between low and high frequency cutoffs. Our ALS had the bandwidth of 133 Hz–6.52 kHz. THD was measured at three different input frequencies. At the input of 500 Hz and 70 dB (SPL), THD was 11.8%. At the input of 800 Hz and 70 dB (SPL), THD was 13.3%. At the input of 1,600 Hz and 65 dB (SPL), THD was 2.5%. Reading from the results, it can be seen that the ALS has a gain of 67.3 dB, which is considerably high as intended, comparable to the rage of hearing aid amplification needed for user with moderate-to-severe hearing loss. The bandwidth achieved also covers the targeted speech rage. In terms of THD, the values are relatively high compared to the range of 1-2% expected from hearing aids, but still competitive compared to THD of FM products available in the markets that we also measured. This implies that the quality of the audio signal would be between that of hearing aids and commercial FM products.

#### 3.2.2. Field Test

The field test was designed to test our ALS in an actual operating environment which was in classrooms of hearing-impaired students. It was also tested by prospective users, which were the hearing-impaired students from our partner school. In the test, students would listen to separate Thai words and write them down. The results would reflect their ability to hear and recognise words when using ALS.

For comparison, each student would do three identical tests but using three different devices. They would wear our ALS, a commercial FM ALS currently used in the school, and their own hearing aids with no ALS. The test details were as follows.

- It was carried out in the school library. The recorded words were played from a speaker. The testers sat 10 metres away from the speaker. The library was used because available classrooms were 8 metres long, which were shorter than our ALS specifications.
- Five students participated in the test. All of them had severe (71–90 dB HL) to profound (91+ dB HL) levels of hearing loss of both ears [9]. They were all in primary school year five and six. They were all taught orally, without using sign language at school.
- Three sets of 25 phonetically balanced (PB) Thai words were used, one for each test.
- Each word was played three times. Students would pronounce the word they hear and write it down.
- Each student was accompanied by an observer who would listen and note down the words each student said. This was because students may have limited spelling skills. They might have heard the word correctly but spelt it wrongly. The observers' notes would be used for analysis.
- The order of word sets and devices used was randomly set for each student.
- During the test, SNR was measured to be between 10 and 15.
- Classification of hearing correctness was listed as follows:
  - correct absolutely (whole word);
  - correct only for the first syllable and vowel. English equivalent example of this case would be hearing “pass” instead of “past”;
  - correct only for the first syllable. English equivalent example of this case would be hearing “cat” instead of “cow”;
  - correct only for the vowel. English equivalent example of this case would be hearing “can” instead of “bat”;
- Class teachers would remove the words, perceived to be unknown to individual students from the result.

**Figure 7:**
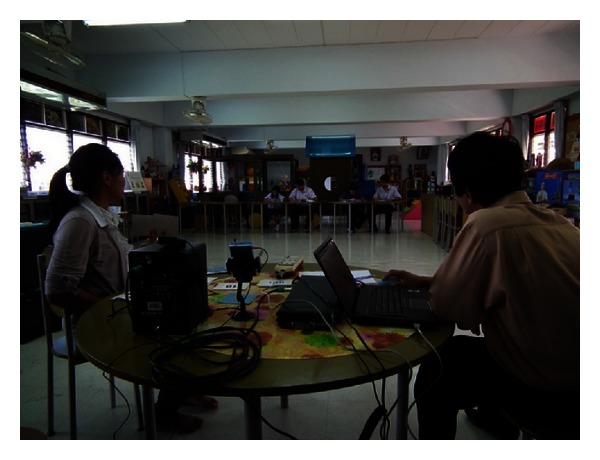
Field test.

The experiment was shown in Figure 7.

*(a) Field Test Results.* The raw results for individual students are shown in Table 1. Summarised results in percentage are shown in Table 2.

*(b) Field Test Result Analysis*. From Table 2, our ALS receives approximately 6% higher whole word correctness than school ALS. Overall correctness percentage of school ALS is higher than our ALS by approximately 2.5%. These results suggest that our and school ALSs performance is similar.

It should be noted that hearing performance of students when wearing either our or school ALS is nearly twice as much as when not wearing ALS. The result supports the obvious benefit of ALS to hearing-impaired people. At 10 metres, original audio signal deteriorates so much that makes hearing difficult. ALS helps transmit signal while maintaining quality close to the original signal.

It can be seen from Table 1 that hearing ability of students varies greatly, even though they study at a comparable level. Student 5 got 43 whole words correct, while student 4 only got 15 correct. One teacher added that recognising separate words is difficult for hearing-impaired students. The students had better understanding of sentences through context cognition.

The notes from the observers are not reported here, because the observers were mostly unable to recognise the words that students pronounced. It is observed that only skillful listeners such as teachers could understand individual students' speech.

## 4. Conclusions

In this work, we describe the development of a low cost wireless broadcasting ALS. It was designed for short-range application, such as in the classroom for the hearing-impaired students. The results from audio characteristics and field test, by prospective users, suggest that its performance is comparable to an existing ALS system. It is hence a viable alternative to existing FM ALS. 

The ALS was also designed to address another practical issue commonly faced when students usually found using hearing aids that are incompatible with existing ALS (i.e., no neckloop or DAI shoe-in), making them either not using the ALS or using it without the hearing aid. The ALS in this project was designed with a built-in amplifier to help the students to have the choice of using ALS alone without completely losing hearing aid's amplification benefits. The built-in amplifier was shown to have a gain of 67.3 dB, a level that can be useful for students with moderate-to-severe hearing loss, with the bandwidth that can appropriately support speech communication. 

The bill of materials of the system costs approximately 150 US dollars for each transmitter or receiver. Our system costs several times less than the commercial FM ALS currently used in our partner school.

Our ALS exceeds initial power target of lasting for four hours. It contains one 3.7 V 1000 mA lithium ion battery. When fully charged, it can operate up to ten hours. In school usage, students can wear them in class all day, and the teachers can charge them overnight.

The 2.7 cm × 7.8 cm × 2.7 cm dimension makes our ALS fit into student's shirt pocket or snap to teacher's belt comfortably. Each unit weighs just over 100 grams. We anticipate that it could be made lighter, by changing plastic material for casing. The current material is PVC which is strong but heavy.

## 5. Further Work

We are in the process of assembling a set of two transmitters and ten receiver units. They would be given to our partner school for long-term use. We plan to keep usage and maintenance record for at least six months. This should provide data of our ALS robustness and durability, which is our main concern. From observation there seems to be much wear and tear with children users. Furthermore, there are additional chances of the system being accidentally exposed to moisture and water. This would severely test our system robustness and durability.

## Figures and Tables

**Table 1:** Field test results by students.

Correctness results	Student 1			Student 2			Student 3			Student 4			Student 5		
	No ALS	Our ALS	School ALS	No ALS	Our ALS	School ALS	No ALS	Our ALS	School ALS	No ALS	Our ALS	School ALS	No ALS	Our ALS	School ALS
Whole word	10	12	6	3	11	14	3	8	17	7	4	4	7	24	12
First syllable and vowel	0	0	0	0	0	0	0	0	0	0	0	1	0	0	0
First syllable only	0	0	0	0	0	0	0	0	0	0	0	0	0	0	0
Vowel only	0	2	2	1	0	3	1	2	1	0	0	2	1	0	5
Unknown word(s)	4	2	2	2	4	2	4	2	2	0	0	0	0	0	0

**Table 2:** Summarised results.

Correctness percentage	No ALS	Our ALS	School ALS
Whole word	26.09	50.43	44.54
First syllable and vowel	0	0	0.84
First syllable only	0	0	0
Vowel only	2.61	3.42	10.92
